# The Mechanism of Action of Ursolic Acid as a Potential Anti-Toxoplasmosis Agent, and Its Immunomodulatory Effects

**DOI:** 10.3390/pathogens8020061

**Published:** 2019-05-09

**Authors:** Won Hyung Choi, In Ah Lee

**Affiliations:** 1 Marine Bio Research & Education Center, Kunsan National University, 558 Daehak-ro, Gunsan-si, Jeollabuk-do 54150, Korea; 2 Department of Chemistry, College of Natural Science, Kunsan National University, 558 Daehak-ro, Gunsan-si, Jeollabuk-do 54150, Korea; leeinah@kunsan.ac.kr

**Keywords:** parasite, toxoplasmosis, microneme, rhoptry, immune activity, anti-inflammation

## Abstract

This study was performed to investigate the mechanism of action of ursolic acid in terms of anti-*Toxoplasma gondii* effects, including immunomodulatory effects. We evaluated the anti-*T. gondii* effects of ursolic acid, and analyzed the production of nitric oxide (NO), reactive oxygen species (ROS), and cytokines through co-cultured immune cells, as well as the expression of intracellular organelles of *T. gondii*. The subcellular organelles and granules of *T. gondii*, particularly rhoptry protein 18, microneme protein 8, and inner membrane complex sub-compartment protein 3, were markedly decreased when *T. gondii* was treated with ursolic acid, and their expressions were effectively inhibited. Furthermore, ursolic acid effectively increased the production of NO, ROS, interleukin (IL)-10, IL-12, granulocyte macrophage colony stimulating factor (GM-CSF), and interferon-β, while reducing the expression of IL-1β, IL-6, tumor necrosis factor alpha (TNF-α), and transforming growth factor beta 1 (TGF-β1) in *T. gondii*-infected immune cells. These results demonstrate that ursolic acid not only causes anti-*T. gondii* activity/action by effectively inhibiting the survival of *T. gondii* and the subcellular organelles of *T. gondii*, but also induces specific immunomodulatory effects in *T. gondii*-infected immune cells. Therefore, this study indicates that ursolic acid can be effectively utilized as a potential candidate agent for developing novel anti-toxoplasmosis drugs, and has immunomodulatory activity.

## 1. Introduction

Recently, zoonotic diseases have been consistently occurring in different countries worldwide, causing serious threats in many countries and to humans globally. In this respect, the prevention and education of various zoonotic diseases are becoming a major issue. Toxoplasmosis is a parasitic disease that is induced through the infection of *Toxoplasma gondii* in many countries worldwide as a zoonotic parasitosis, causing serious diseases and chronic infection in various sites of the human body in all age groups, including adults and young children as well as pregnant women. In particular, because toxoplasmosis is a zoonotic disease that infects humans through cats, many people that have cats as pets, particularly pregnant women, need to take caution in this regard. In the biological aspect, *T. gondii* not only has intracellular organelles such as the golgi, endoplasmic reticulum, and mitochondrion, but also unique subcellular organelles such as the conoid, apicoplast, surface antigens (SAGs), dense granule proteins (GRAs), rhoptries, and micronemes [1]. *T. gondii* has an inner membrane complex (IMC) involving the plasma membrane, consisting of a unique double membrane structure which is combined with a cytoskeletal network. The IMC acts as a major factor in the proliferation and growth for the survival of *T. gondii*, as well as in motility and cell division, which has been reported to include different sub-proteins such as IMC sub-compartment proteins 1, 2, and 3 [2,3,4]. The protein kinases of *T. gondii* have been known to play key roles for modulating the motility, invasion, replication, egress, and survival within the host [5]. It was reported that *T. gondii* is divided through a unique form of cell division such as endodyogeny, and its chromosomal passenger complex is essential for the organization of a functional mitotic spindle [6]. Furthermore, *T. gondii* not only forms a parasitophorous vacuole membrane (PVM) after invasion into host cells, but also proliferates and grows into a PVM [7,8,9]. For decades, several compounds through many researchers and groups were developed as drugs for treating toxoplasmosis, and are usefully used in clinics. However, existing drugs are widely vulnerable to drug-resistance in clinics globally. To overcome pharmacological barriers to these challenges, the efforts for treating infectious diseases have been attempted in various fields, and it has been reported that various extracts/compounds derived from medicinal plants and new synthetic compounds have anti-*T. gondii* effects in the in vitro and pre-clinic stages [10,11,12,13,14,15]. Until recently, various compounds derived from plants have showed a leading role and ability as a source of specialized metabolites with medical effects as well as pharmacological activities. In addition, it was reported that various extracts/compounds derived from plants and synthetic compounds could be used in the medical field as useful resources for treating acute or chronic infectious diseases caused by schistosomiasis, leishmania, malaria, or tuberculosis, as well as viral diseases including middle east respiratory syndrome (MERS) or Zika fever and avian influenza [16,17,18,19,20,21,22,23].

However, effective next-generation drugs for treating toxoplasmosis have not yet been developed, and the difficulty of drug development against zoonosis is still causing a public health crisis globally. In this context, various studies on the development or discovery of effective drugs and novel candidates against parasitic zoonosis are urgently required. In this regard, ursolic acid is a bioactive compound derived from medicinal plants, and is also known to have selective bioactive properties such as anti-inflammatory [24,25,26,27] and anticancer effects [28,29,30,31]. It also effectively induces various activities, including antimicrobial [32,33,34] and anti-parasitic effects [35,36]. Until recently, although studies and compounds regarding anti-*T. gondii* activity have been reported globally [37,38,39,40,41,42,43], the mechanism of action of ursolic acid on the inhibitory effect of *T. gondii* and the immunomodulatory activity was not reported yet. From this perspective, this study was performed to evaluate the mechanism of action and the immunomodulatory activity of ursolic acid in terms of anti-*T. gondii* effects and activity, and to confirm the potential as a potent candidate drug for developing novel anti-toxoplasmosis agents.

## 2. Results

### 2.1. Anti-Parasitic Effect of Ursolic Acid Against the Viability of T. gondii

**Figure 1:**
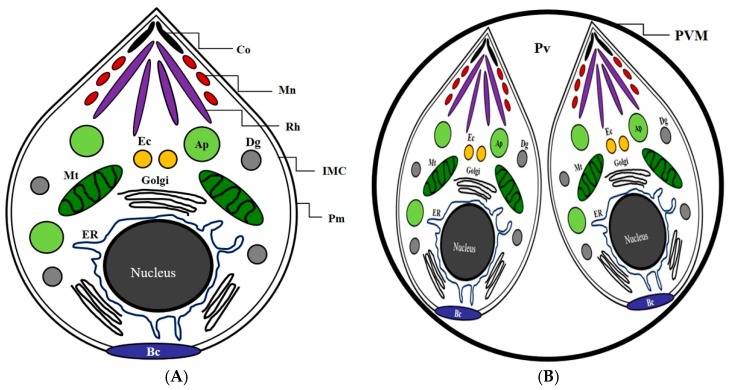
The basic structure and configuration diagram of *Toxoplasma gondii*. (**A**) The basic structure and subcellular organelles of *Toxoplasma gondii*. (**B**) *T. gondii* in the intracellular parasitophorous vacuole membrane of a host cell. Co: conoid; Mn: microneme; Rh: rhoptry; IMC: inner membrane complex; Pm: plasma membrane; Ap: apicoplast; Dg: dense granule; Mt: mitochondrion; ER: endoplasmic reticulum; Ec: endosome compartment; Bc: basal complex; Pv, parasitophorous vacuole; PVM, parasitophorous vacuole membrane.

**Figure 2:**
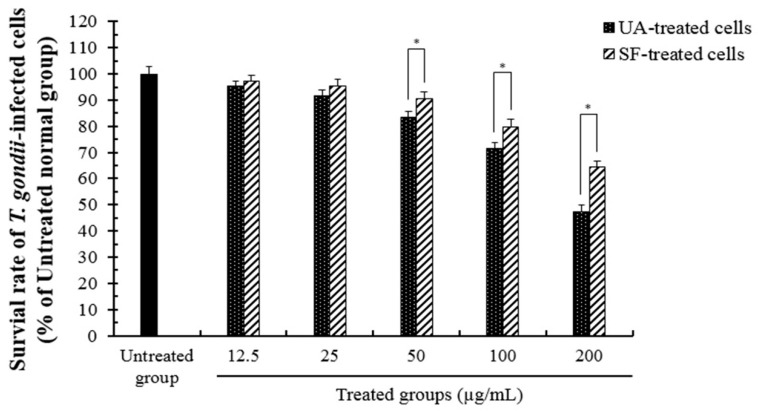
The inhibitory effect of ursolic acid against the survival of *T. gondii*-infected lung cells. The normal lung cells were infected with *T. gondii* (cells: *T. gondii* = 1:4), and *T. gondii*-infected cells were treated with different concentrations (12.5–200 μg/mL) of ursolic acid (UA) and sulfadiazine (SF) at 37 °C for 24 h, respectively; their survival rates were inhibited a dose-dependent manner. The results are presented as a percentage of the untreated normal group. * *p* < 0.05 was considered to be significant.

*T. gondii* has a specific network structure and systems, including various intracellular organelles such as the mitochondrion, apicoplast, dense granule, and rhoptry (Figure 1), which induces serious zoonotic diseases such as toxoplasmosis, particularly in humans, while causing infection in various vertebrates. Before evaluating the mechanism of action of ursolic acid against *T. gondii*, we measured the inhibitory effect of ursolic acid (UA) against the survival of *T. gondii* and normal lung cells infected with *T. gondii* through an MTT assay as described previously [9], which showed significant results and reproducibility. When *T. gondii* was treated with UA (12.5–400 μg/mL) for 24 h, the viability was effectively inhibited, and the parasitic survival rate was measured as less than 30% at 200 μg/mL. UA strongly decreased the viability of *T. gondii* compared with sulfadiazine (SF), which demonstrates that UA caused anti-*T. gondii* effect or activity against *T. gondi* (data not shown). In particular, the lung cells infected with *T. gondii* were markedly inhibited in a dose-dependent manner after treatment with ursolic acid (12.5–200 μg/mL) when compared with the untreated infection cells and the *T. gondii*-infected cells treated with SF (Figure 2). Furthermore, it was clearly shown that there were significant differences between the untreated *T. gondii*-group and the experimental groups. In these aspects, it was demonstrated that UA effectively induced the selective anti-parasitic effect/action compared to the SF-treated group and the untreated group through a direct inhibitory effect on the viability of *T. gondii*. This shows that UA has the potential to be utilized as an anti-*T. gondii* candidate agent and/or a synergic compound with the existing drugs for developing novel anti-toxoplasmosis agents. Therefore, these results indicate that UA strongly inhibits or blocks the survival of *T. gondii* by effectively inducing anti-*T. gondii* activity through the selective inhibitory activity and/or action against the viability of *T. gondii*.

### 2.2. The Inhibitory Effects of Subcellular Organelles of T. gondii

**Figure 3:**
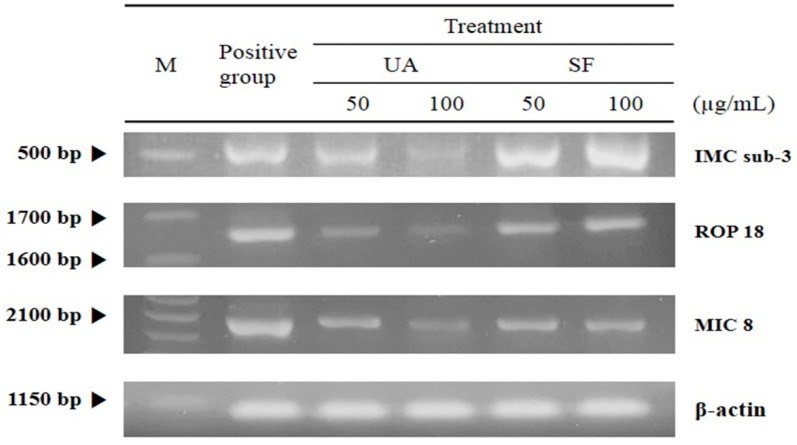
The change of the expression of the intracellular organelles of *T. gondii* in *T. gondii*-infected cells. The normal lung cells were infected with *T. gondii* (cells: *T. gondii* = 1:4), and *T. gondii*-infected cells were incubated with 50 and 100 μg/mL of ursolic acid (UA) and sulfadiazine (SF) at 37 °C for 24 h, respectively. Total RNA was isolated from *T. gondii* strain using RNeasy Mini Kit, and the intracellular organelles of *T. gondii* (rhoptry protein 18, microneme protein 8, and inner membrane complex sub-compartment protein 3) were synthesized to complementary DNA (cDNA) through one-step RT-PCR kit. The specific genes were compared through the untreated positive group and the treated group. M: DNA marker.

*T. gondii* causes unique anti-apoptotic features and parasitic survival cycles or stages, such as the inactivation of apoptotic proteins during the parasitic life-cycle in host cells after infection, showing critical roles during the proliferation and survival of *T. gondii.* In particular, the PVM, that functions as a critical indicator during the proliferation stage of *T. gondii*, is activated in a time-dependent manner after cell invasion of *T. gondii*, which causes finally the proliferation and the growth of *T. gondii.* In this aspect, we investigated the inhibitory action of UA against subcellular organelles such as rhoptries, micronemes, and inner membrane complex that form its intracellular metabolic network as well as the homeostasis of *T. gondii*. As shown in Figure 3, when *T. gondii* was treated with different concentrations (50 and 100 μg/mL) of UA and SF, respectively, the intracellular organelles (rhoptry protein 18 (ROP 18), microneme protein 8 (MIC 8), and inner membrane complex sub-compartment protein 3 (IMC sub-3)) of *T. gondii* were markedly decreased in a dose-dependent manner, and their changes were clearly observed under electrophoresis. In particular, it is known that rhoptry plays as a critical factor when *T. gondii* enters into host cells during invasion stage [44,45,46,47,48]. In these aspects, this result shows clearly that UA not only has the direct inhibitory effect against the survival of *T. gondii* but also induces the anti-*T. gondii* effect by strongly blocking or inhibiting the homeostasis and network structure of the intracellular organelles of *T. gondii.* Therefore, these results demonstrate substantial evidence that UA consistently causes anti-*T. gondii* activity/action by effectively inhibiting the intracellular organelles of *T. gondii*. 

### 2.3. The Effect of Reactive Oxygen Species (ROS) and Nitric Oxide (NO) Production of Ursolic acid in T. gondii-Infected Cells

**Figure 4:**
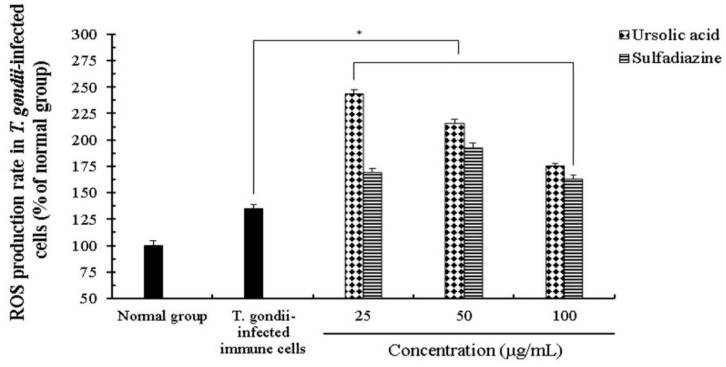
The effect of reactive oxygen species (ROS) production in *T. gondii*-infected immune cells treated by compounds. The co-cultured cells (macrophage and basophil cells) were infected with *T. gondii* (cells: *T. gondii* = 1:4), and the cells were incubated with different concentrations (25–100 μg/mL) of ursolic acid (UA) and sulfadiazine (SF) at 37 °C for 24 h, respectively. The results are presented as a percentage of the normal group. * *p* < 0.05 was considered to be significant.

**Figure 5:**
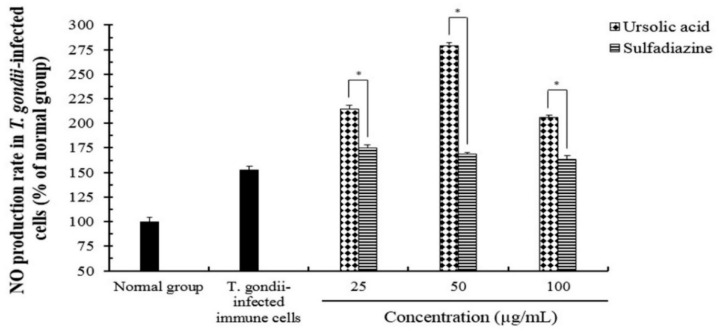
The effect of nitric oxide (NO) production in *T. gondii*-infected immune cells treated by compounds. The co-cultured cells (macrophage and basophil cells) were infected with *T. gondii* (cells: *T. gondii* = 1:4), and the cells were incubated with different concentrations (25–100 μg/mL) of ursolic acid (UA) and sulfadiazine (SF) at 37 °C for 24 h, respectively. The results are presented as a percentage of the normal group. * *p* < 0.05 was considered to be significant.

We evaluated the effect of ROS and NO production induced by ursolic acid in immune cells infected with *T. gondii*. The production of NO and ROS was measured in a dose-dependent manner when *T. gondii*-infected co-cultured immune cells were incubated with various concentrations (25–100 μg/mL) of UA and SF for 24 h, respectively. In particular, the production of ROS was increased in the *T. gondii*-infected group treated with UA compared with *T. gondii*-infected group. However, in 100 μg/mL of UA, ROS activity gradually indicated lower concentrations than other UA-treated groups (Figure 4), and these changes of ROS activity show that UA acts as a modulator which consistently inhibits the proliferation and the growth of *T. gondii* by promoting the production of ROS in immune cells after the parasite infection. Even though SF-treated groups significantly increased ROS production compared to the *T. gondii*-infected group, there were low ROS-productive rates compared to UA-treated groups. Furthermore, the rate of NO production was strongly increased in *T. gondii*-infected group treated with UA compared with normal and *T. gondii*-infected groups, but in the range of 100 μg/mL of UA, the rate of NO production were low concentrations compared with other UA-treated groups (Figure 5). However, the activities of both ROS and NO induced by UA were significantly increased more than normal and infection groups. Interestingly, the activities of ROS and NO in *T. gondii*-infected cells treated with SF were lower than *T. gondii*-infected groups treated with UA. The activities of ROS and NO were significantly increased in *T. gondii*-infected groups treated with UA and SF, respectively, whereas their activities were gradually reduced in high concentrations. These results show that UA not only consistently induces the production of ROS and NO through the interaction between it and immune cells in *T. gondii*-infected immune cells, but may also be used as a synergic compound for the immunomodulation after parasitic infection.

### 2.4. The Change of Cytokines by Interaction of Ursolic Acid and Immune cells

**Figure 6:**
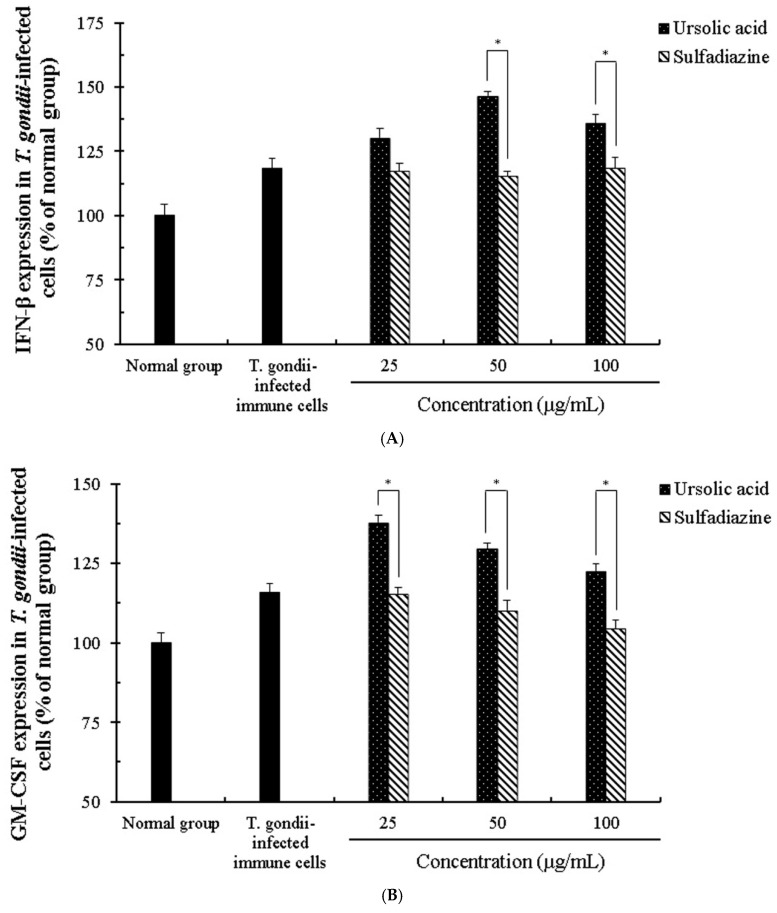
The production rates of interferon-β and granulocyte macrophage colony stimulating factor (GM-CSF) in *T. gondii*-infected immune cells. The co-cultured cells immune cells were infected with *T. gondii* (cells: *T. gondii* = 1:4), and *T. gondii*-infected immune cells were incubated with different concentrations (25–100 μg/mL) of ursolic acid (UA) and sulfadiazine (SF) at 37 °C for 24 h, respectively. (**A**) the interferon-β (IFN-β) group and (**B**) GM-CSF group. The results are presented as a percentage of the normal group. * *p* < 0.05 was considered to be significant.

**Figure 7:**
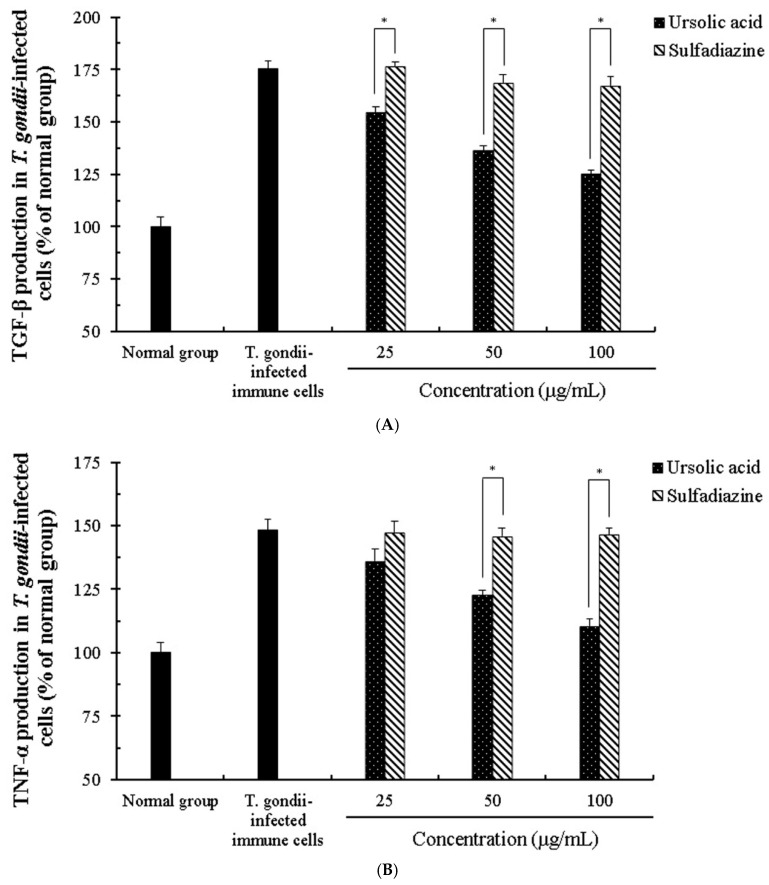
The change of production of transforming growth factor beta 1 (TGF-β1) and tumor necrosis factor alpha (TNF-α) in *T. gondii*-infected immune cells. The co-cultured cells immune cells were infected with *T. gondii* (cells: *T. gondii* = 1:4), and *T. gondii*-infected immune cells were incubated with different concentrations (25–100 μg/mL) of ursolic acid (UA) and sulfadiazine (SF) at 37 °C for 24 h, respectively. (**A**) TGF-β1 group and (**B**) TNF-α group. The results are presented as a percentage of the normal group. * *p* < 0.05 was considered to be significant.

*T. gondii* proliferates rapidly in host cells through parasitic interactions and signals for its survival after cell invasion, which finally induces destruction of host cells. We evaluated the synergic effect and the activation of cytokines caused by ursolic acid in co-cultured immune cells (macrophages, T cells, B cells and basophil cells) infected with *T. gondii*. The production of the cytokines (interferon-β, granulocyte macrophage colony stimulating factor (GM-CSF), transforming growth factor beta 1 (TGF-β1), and tumor necrosis factor alpha (TNF-α)) in *T. gondii*-infected immune cells was notably changed in a concentration-dependent manner when the cells were incubated with different concentrations (25–100 μg/mL) of UA, and their changes were measured under an ELISA reader. In particular, the production of interferon-β and GM-CSF was effectively increased in *T. gondii*-infected cells treated with UA compared with *T. gondii*-infected cells, and these changes show that UA accelerated the production and release of interferon-β and GM-CSF by stimulating the macrophage, B cells, and basophil cells (Figure 6). Moreover, the production of interferon-β was not changed in SF-treated groups, and the production of GM-CSF was gradually decreased. Furthermore, the expression of TGF-β1 and TNF-α was markedly reduced in *T. gondii*-infected cells treated with UA, whereas *T. gondii*-infected cells treated with SF did not cause the production of TGF-β1 and TNF-α compared with other groups (Figure 7). Interestingly, *T. gondii*-infected cells treated with SF did not significantly produce the expression of Interferon-β, TGF-β1 and TNF-α compared to the infected group. This shows that SF is not involved in immune response for expressing TNF-α, TGF-β1, and interferon-β in immune cells. Furthermore, the production of TGF-β1 was decreased in *T. gondii*-infected cells treated with UA, but the result shows that UA reduced the expression of TGF-β1 in immune cells by directly inhibiting *T. gondii*. These results demonstrate that UA not only promotes defense against parasitic infection by effectively inducing the production of Interferon-β and GM-CSF from immune cells when the cells are infected with *T. gondii*, but also inhibits the overexpression of inflammatory cytokine by reducing the expression of TNF-α caused by infection.

### 2.5. The Activity and Change of Cytokines by Stimulation of Ursolic acid

**Figure 8:**
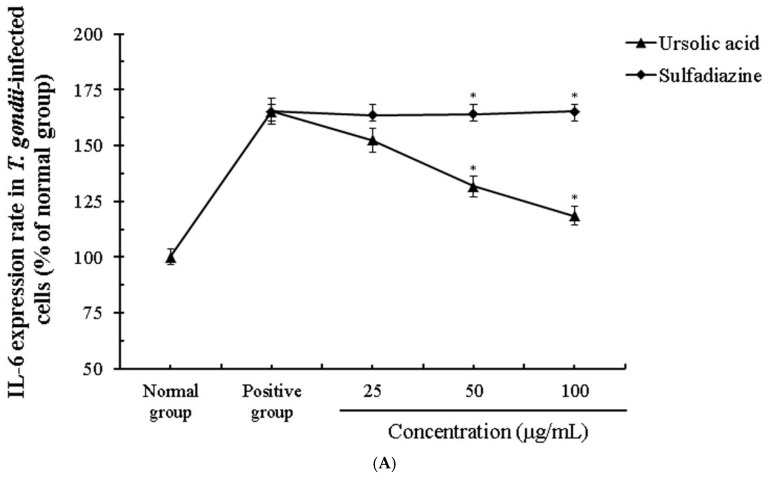

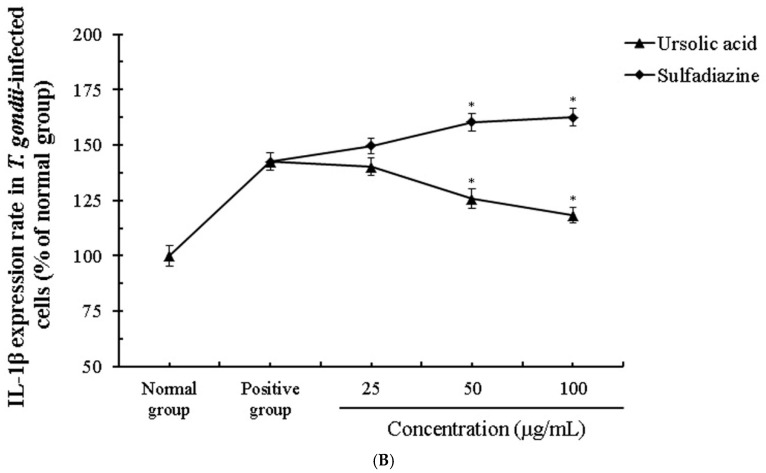
The change of the expression of interleukin (IL)-1β and IL-6 in *T. gondii*-infected immune cells. The co-cultured cells immune cells were infected with *T. gondii* (cells: *T. gondii* = 1:4), and *T. gondii*-infected immune cells were incubated with different concentrations (25–100 μg/mL) of ursolic acid (UA) and sulfadiazine (SF) at 37 °C for 24 h, respectively. (**A**) IL-6 group and (**B**) IL-1β group. The results are presented as a percentage of the normal group. * *p* < 0.05 was considered to be significant.

**Figure 9:**
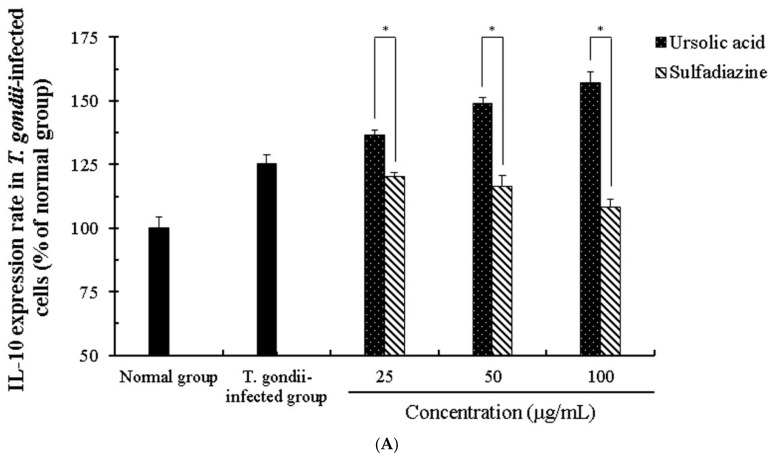

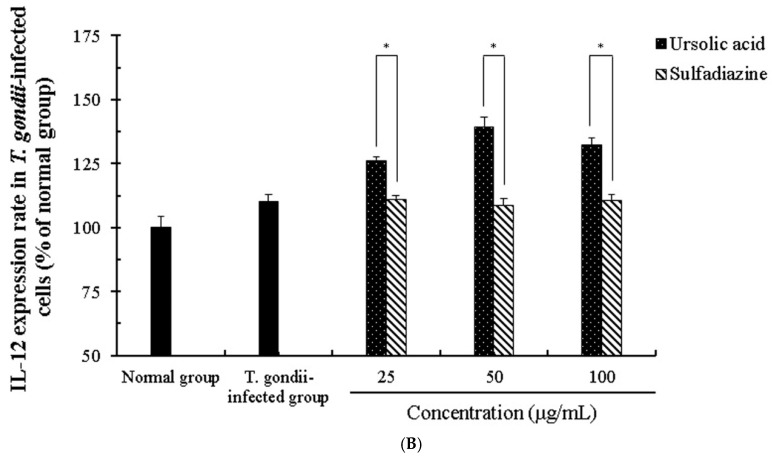
The activity of IL-10 and IL-12 in *T. gondii*-infected immune cells. The co-cultured cells immune cells were infected with *T. gondii* (cells: *T. gondii* = 1:4), and *T. gondii*-infected immune cells were incubated with different concentrations (25–100 μg/mL) of ursolic acid (UA) and sulfadiazine (SF) at 37 °C for 24 h, respectively. (**A**) IL-10 group and (**B**) IL-12 group. The results are presented as a percentage of the normal group. * *p* < 0.05 was considered to be significant.

We evaluated the activation and interaction of various cytokines induced by ursolic acid through co-cultured immune cells (macrophage, T cells, B cells and basophil cells) infected with *T. gondii*. The changes of the cytokines (interleukin (IL)-1β, IL-6, IL-10, and IL-12) in *T. gondii*-infected immune cells were measured in a dose-dependent manner when the cells were incubated with different concentrations (25–100 μg/mL) of UA, and their changes were evaluated under an ELISA reader. In particular, the production of IL-1β and IL-6 was reduced in the groups treated with UA compared with the infection group, and the expression of IL-10 and IL-12 was significantly increased in *T. gondii*-infected cells treated with UA (Figure 8 and Figure 9). These changes demonstrate that UA promotes the production and the expression of IL-10 and IL-12 by consistently stimulating the immune cells as well as inhibit the expressions of IL-1β and IL-6 induced by *T. gondii* infection. Interestingly, the production of IL-1β in *T. gondii*-infected cells treated with SF was significantly increased and the expression of IL-10 was gradually decreased compared with the infection group. However, there were no significant changes of the production of IL-6 and IL-12 in *T. gondii*-infected cells treated with SF. In particular, significant differences in the cytokines were clearly measured between the *T. gondii*-infected group and UA-treated group, and these changes of cytokines were confirmed in all the groups. Furthermore, the results show that this co-culture system through immune cells can be usefully utilized as a co-culture method for measuring various cytokines, and as an effective alternative to in vivo experiments. These results indicate that UA not only effectively induces the production of cytokines between the immune cells by increasing the expression of IL-10 and IL-12 in macrophage and B cells after *T. gondii* infection, but also suppresses or strongly reduces the expression of the inflammatory cytokines from the immune cells. 

## 3. Discussion

In spite of constant public health efforts worldwide for the past decades, infectious diseases such as malaria, influenza, cholera, yellow fever, MERS, SARS, and tuberculosis have been consistently causing crises in public health as well as global concerns due to their pandemic potential. These serious infectious pathogens have enhanced adaptability to environmental variation and infectivity to host as well as resistance to existing drugs for their viability, evolving in a time-dependent manner. In these aspects, global nonprofit organizations and/or profit companies such as WHO and the Bill & Melinda Gates Foundation as well as various global leading pharmaceutical companies have continuously supported the study for blocking or treating chronic infectious diseases such as Zika, AIDS, and tuberculosis as well as acute infectious diseases including influenza, malaria, cholera, yellow fever, and Ebola, focusing on the development of novel drugs and effective vaccine against pathogens. Furthermore, in spite of various efforts for developing anti-parasitic drugs against zoonotic parasitosis, effective novel drugs such as anti-malaria drugs have not yet been developed or launched. Many people are living with animals such as cats and dogs. In particular, toxoplasmosis, a zoonotic parasitosis, is transmitted via physical contact with pets and companion animals, including cat and dogs.

With respect to these aspects, we carefully focused on the parasitic infectious diseases that are consistently caused through various infection pathways and factors. Among various parasites, *T. gondii* induces serious symptoms such as cerebral calcification and meningoencephalitis in the brain, while causing fetal infection through mother during pregnancy, as it is a zoonotic parasite which affects both humans and animals, resulting in parasitic diseases such as toxoplasmosis [49,50]. Furthermore, it induces serious complications in immune-deficient patients and HIV patients, which attenuates the interaction of the immune system that specifically responds to pathogens in the host [51,52]. *T. gondii* inhibits the interaction of cytokines such as IL-4 and IFN-γ that are activated through the immune system, attenuating the production of cytokines from host cells. In addition, it is known that *T. gondii* blocks parasitic inhibitory system while inhibiting the signal pathways of cell cycle initiators or apoptotic mediators for the apoptotic stage in host cells after invasion [53,54]. *T. gondii* infection induces an imbalance of immune response by causing changes in cytokines such as TNF-α and TGF-β1 in the host, resulting in immune deficiency by breaking homeostasis and interaction of the immune system in the host. Moreover, *T. gondii* induces the proliferative and growth cycle of *T. gondii* by rapidly forming PVM and by regulating cell cycle factors for its survival in host cells, maintaining the survival by accelerating the proliferation of *T. gondii* in host [55,56]. The subcellular organelles and granules of *T. gondii*, such as the conoid, GRAs, SAGs, the rhoptry, microneme proteins, and the inner membrane complex, were focused upon as major targets for blocking *T. gondii* infection, which was used as major recombinant proteins or factors for protective immunity including vaccination [57,58,59,60]. For these reasons, in this study we not only evaluated the anti-parasitic effect of UA which inhibits the survival of parasite after *T. gondii* infection through a *T. gondii*-infected co-cultured immune system, but also confirmed its mechanism of action with respect to anti-*T. gondii* effects and immunomodulatory activity. In the present study, the survival rates of *T. gondii* were markedly inhibited when the parasites were treated with UA and SF compared with the non-treated *T. gondii* group, and the expression of subcellular organelles and granules of *T. gondii* in the infected cells was effectively decreased in *T. gondii*-infected group treated with UA compared with other groups. Furthermore, the production of ROS and NO was significantly increased in the *T. gondii*-infected group treated with UA compared with both SF-treated and infection groups. Interestingly, although ROS production was activated in both *T. gondii*-infected cells treated with UA and SF, its rate was gradually decreased in the range of high concentrations of the compounds. In these aspects, NO and ROS production not only activates immune defense systems against various pathogens such as bacteria and viruses but also maintains effectively the changes of the homeostasis in the organism, promoting the production of cytokines induced by the interaction of immune cells. Cytokines such as IL-1β, IL-6, and TNF-α were increased in the infected cells after *T. gondii* infection, and their production was significantly suppressed after UA treatment. In particular, although the production of TGF-β1 was reduced in *T. gondii*-infected immune cells treated with UA, it was shown that UA suppressed the expression of TGF-β1 in immune cells by inhibiting the survival of *T. gondii*. In addition, UA not only effectively increased the production of anti-inflammatory cytokines such as IL-10 and interferon-β in *T. gondii*-infected cells but also activated the expression of IL-12 and GM-CSF, promoting the development of Th1 cells and the production of interferon-γ that is induced by NK cells.

Taken together, the results of this study showed the anti-parasitic effects of UA against the survival of *T. gondii* as well as demonstrated its mechanism of action causing anti-*T. gondii* effect in *T. gondii*-infected co-cultured cells. Furthermore, these results indicated that the expression of subcellular organelles and granules of *T. gondii* was specifically inhibited or blocked in a concentration-dependent manner after treatment with UA. This study provides evidence that UA not only decreases the inflammatory cytokines (IL-1β, IL-6 and TNF-α) expressed from the immune cells after *T. gondii* infection, but also effectively increases the production of anti-inflammatory mediators such as IL-10, IL-12, GM-CSF, and interferon-β, including the activation of ROS and NO production. Interestingly, it was confirmed in this study that macrophage and other immune cells did not directly inhibit or rapidly decrease the survival of parasite as well as the proliferation of *T. gondii* through the bactericidal action such as phagocytosis of macrophage after *T. gondii* infection. This may be the result of the differences between the in vitro system and the in vivo experiment including rats and mice. However, the parasite may not be eliminated by the bactericidal action of macrophages and other immune cells in the infected body because *T. gondii* is found in various body sites of patients in the clinic. Moreover, it may be changed or maintained to the parasite type that indicates characteristics of hibernation like *T. gondii* ME49 strain (type II) in the body.

In summary, the results of this study demonstrate that UA induces anti-*T. gondii* effects/action by effectively blocking or inhibiting the survival of *T. gondii*, and also has anti-parasitic activity that consistently inhibits the proliferation/growth of *T. gondii* by strongly inhibiting the expression of subcellular organelles and granules of *T. gondii*, such as ROP 18, MIC 8, and IMC sub 3 in *T. gondii*-infected cells. In addition, these results show clearly that UA has the immunomodulatory action/activity which causes the change of cytokines in immune cells by decreasing the inflammatory cytokines (IL-1β, IL-6, and TNF-α) and/or by activating anti-inflammatory cytokines including IL-10, IL-12, GM-CSF, or interferon-β through the interaction between UA and immune cells. Furthermore, the results indicate that UA induces the death of *T. gondii* by promoting the production of ROS and NO, such as super oxides and hydroxyl radicals in *T. gondii*-infected immune cells. Therefore, this study indicates that UA can be used effectively as a potent candidate agent or a synergic compound with existing drugs in the development of novel anti-*T. gondii* drugs of the next generation against toxoplasmosis, with potential as a modulatory substance against the immune response of the parasite in parasite-infected immune cells. Moreover, UA requires further study for efficacy and safety against toxoplasmosis in preclinical stages through in vivo animal models in the near future.

## 4. Experimental Section

### 4.1. Materials

RPMI medium 1640, Dulbecco’s modified eagle medium (DMEM), fetal bovine serum (FBS), phosphate buffered saline (PBS), antibiotics, and trypsin-EDTA solution were purchased from Invitrogen Corporation (Gibco®, U.S.A). MTT (3-(4,5-dimethylthiazol-2-yl)-2,5-diohenyl-2H-tetrazolium bromide; thiazolyl blue), dimethyl sulfoxide (DMSO), 0.4% trypan blue, and sulfadiazine were purchased from Sigma-Aldrich Chemical Co., Ltd. (St. Louis, MO, U.S.A). All other chemicals and reagents were purchased from Sigma-Aldrich Chemical Co., Ltd. (St. Louis, MO, U.S.A) and Merck Chemical Co., Ltd (Darmstadt, Germany), and Donginbio Co., Ltd. (Seoul, South Korea).

### 4.2. Preparation of Drugs

The anti-toxoplasmosis drug, sulfadiazine (SF), was dissolved in DMSO, and ursolic acid (UA) was also dissolved in DMSO to a concentration of 50 mg/mL according to the manufacturer’s instructions. Sulfadiazine was used as a standard drug to evaluate whether or not ursolic acid has an anti-parasitic effect and activity against *T. gondii*. The compounds were filtered using 0.2-µm membrane syringe filters (Roshi Kaisha, Ltd., Tokyo, Japan) before use, and were stored at −80 °C deep-freezer until use. 

### 4.3. Culture Condition of T. gondii and Cell Lines

Macrophages (Raw 264.7), T cells (EL4), B cells (FB2), basophil cells (RBL-2H3), and normal lung epithelial cells (L2) were purchased from Korean Cell Line Bank at Seoul National University and American Type Culture Collection (Manassas, VA, USA). The cells were cultured in RPMI medium 1640 and DMEM containing 2 mM L-glutamine, supplemented with 15% decomplemented fetal bovine serum (FBS), penicillin (100 units/mL), and streptomycin (100 μg/mL) in a humidified atmosphere containing 5% CO_2_ in air at 37 °C. The *T. gondii* RH was suspended with 1 × PBS, which was injected in the abdominal cavity of each BALB-c/mouse. Five days after the injection, *T. gondii* was collected from the peritoneal fluids of mouse kept in the abdominal cavities of the mice before use. In the in vitro study, host cells were infected with tachyzoite of *T. gondii* at a cell-to-parasite ratio of 1:4.

### 4.4. The Viability of T. gondii and T. gondii-Infected Cells

The inhibitory effect of ursolic acid against *T. gondii* was confirmed by directly evaluating the viability of *T. gondii* exposed to UA and SF through the MTT assay. Briefly, after *T. gondii* was seeded in a 24-well plate (8 × 10^6^/well), *T. gondii* was incubated with different concentrations (12.5–400 μg/mL) of UA and SF for 24 h, respectively. The normal lung cells were infected with *T. gondii* (cells: *T. gondii* = 1:4), and *T. gondii*-infected cells were treated with different concentrations (12.5–200 μg/mL) of ursolic acid (UA) and sulfadiazine (SF) at 37°C for 24 h, respectively; then their viabilities were evaluated by MTT assay. It was to be determined whether or not UA has anti-parasitic activity and/or effect against *T. gondii*. The cells were divided into normal and experimental groups, and the survival rate (SR) of *T. gondii* was calculated as follows: % of SR = (OD_drug-tested wells_ − OD_blank_)/(OD_control_ − OD_blank_) × 100. The optical density (OD) was measured at a wavelength of 570 nm using an ELISA leader. 

### 4.5. RT-PCR Analysis of T. gondii

The inhibitory effect of ursolic acid against the viability of *T. gondii* was evaluated by measuring the expression of subcellular organelles of *T. gondii* in *T. gondii*-infected cells exposed to UA and SF. After normal lung cells were seeded in a 6-well plate (4 × 10^5^/well), the cells were infected with *T. gondii* (1:4, cells/parasite ratio), which was incubated with different concentrations (50–100 μg/mL) of UA and SF for 24 h respectively, and their total RNA was isolated from *T. gondii* strain using RNeasy Mini Kit (Qiagen, Hilden, Germany). Total RNA was obtained from untreated control and treated groups following the manufacturer’s recommended procedure, and the total RNA was quantified in an Eppendorf BioSpectrometer (Eppendorf, Seoul, Korea). One pair of primers was designed using the Primer 3 (version 4.1) and NCBI primer-blast according to the sequence of the selected specific gene in NCBI GenBank as follows: *T. gondii* MIC 8 (accession number: AF353165, 2055 bp), ROP 18 (accession number: AM075204, 1665 bp), IMC sub-3 (accession number: HQ012579, 495 bp), and β-actin (accession number: NM031144, 1128 bp). The specific genes of *T. gondii* were synthesized to complementary DNA (cDNA) through one-step RT-PCR kit (Bioneer, Daejeon, Korea). The cDNA was analyzed by electrophoresis in a 1.2% (w/v) agarose gel containing 10 μg/mL ethidium bromide at 120 V for 1 h, which was visualized under ultraviolet illumination. The β-actin was used as an internal standard for the amount of the expression of cDNA present in each sample. The groups were divided into the untreated positive group (*T. gondii*-infected group) and experimental groups (UA and SF-treated groups).

### 4.6. Determination of NO and ROS Production

The effect of nitric oxide (NO) and reactive oxygen species (ROS) production of ursolic acid was confirmed by measuring NO and ROS production in *T. gondii*-infected co-cultured cells (macrophage and basophil cells) exposed to the compounds. The cells were divided into normal and experimental groups as described above. Briefly, after the co-culture cells were seeded in a 96-well plate (1 × 10^5^/well), the cells were infected with *T. gondii* (1:4, cells/parasite ratio), which was incubated with different concentrations (25–100 μg/mL) of UA and SF for 24 h, respectively. The NO and ROS production were measured in the control and treated groups according to the manufacturer’s instructions through NO and ROS production kits (Cell Biolabs, Inc. San Diego, CA, USA) respectively. The rates of NO and ROS production were measured using an ELISA microplate leader (BioTek Instruments, Inc., Winooski, VT, USA). 

### 4.7. The Cytokine of T. gondii-Infected Immune Cells

The effect of ursolic acid regarding immunomodulatory action was evaluated by measuring the expression of cytokines in *T. gondii*-infected co-culture immune cells (macrophage, T cells, B cells, and basophil cells) exposed to UA and SF, respectively. The cells were divided into normal and experimental groups. First, the macrophage and basophil cells were sequentially seeded in a 6-well plate (1 × 10^5^/well) in a humidified atmosphere containing 5% CO_2_ in air at 37 °C. Second, T cells and B cells were additionally seeded in the plate (1 × 10^5^/well) after 24 h. Namely, the co-cultured immune cells were seeded in a 6-well plate (4 × 10^5^/well) and the cells were finally infected with *T. gondii* (1:4, cells/parasite ratio) after 24 h, and incubated with different concentrations (25–100 μg/mL) of UA and SF for 24 h, respectively. The cytokines (TNF-α, TGF-β1, interferon-β, GM-CSF, IL-1β, IL-6, IL-10, and IL-12) were respectively measured in normal, infected, and treated groups according to the manufacturer’s instruction through cytokine ELISA kits (ThermoFisher Scientific, San Diego, CA, USA) and inflammatory multi-cytokine kits (Qiagen, Hilden, Germany), and the untreated *T. gondii*-infected cells were used as an positive group.

### 4.8. Statistical Analysis

All the results were expressed as mean ± standard deviation (SD) of three independent experiments. Statistical analysis of the data was performed using Student’s *t*-test and analysis of variance (ANOVA). A value of * *p* < 0.05 was considered to be statistically significant.

## Abbreviations

The following abbreviations are used in this manuscript:

GM-CSF	Granulocyte macrophage colony stimulating factor
GRAs	Dense granule proteins
IL-1β	Interleukin 1 beta
IL-6	Interleukin 6
IL-10	Interleukin 10
IL-12	Interleukin 12
IMC sub-3	Inner membrane complex sub-compartment protein 3
MIC 8	Microneme protein 8
NK	Natural killer cell
NO	Nitric oxide
ROP 18	Rhoptry protein 18
ROS	Reactive oxygen species
SAGs	Surface antigens
SF	Sulfadiazine
*T. gondii*	*Toxoplasma gondii*
TGF-β1	Transforming growth factor beta 1
TNF-α	Tumor necrosis factor alpha
UA	Ursolic acid
